# The safety and efficacy of remimazolam tosylate for induction and maintenance of general anesthesia in pediatric patients undergoing elective surgery: Study protocol for a multicenter, randomized, single-blind, positive-controlled clinical trial

**DOI:** 10.3389/fphar.2023.1090608

**Published:** 2023-02-10

**Authors:** Yu-Bo Fang, Cheng-Yu Wang, Yu-Qing Gao, Yu-Hang Cai, Jia Chen, Xu-Lin Zhang, Le-Qi Dong, Wang-Ning Shang-Guan, Hua-Cheng Liu

**Affiliations:** Department of Anesthesiology, Perioperative and Pain Medicine, The Second Affiliated Hospital and Yuying Children’s Hospital of Wenzhou Medical University, Key Laboratory of Anesthesiology of Zhejiang Province, The Second Affiliated Hospital and Yuying Children’s Hospital of Wenzhou Medical University, Wenzhou, Zhejiang, China

**Keywords:** remimazolam, propofol, general anesthesia, pediatric patients, efficacy

## Abstract

**Introduction:** Remimazolam is an ultra-short-acting benzodiazepine sedative agent commonly used in general anesthesia, procedural sedation, and intensive care unit (ICU) sedation. This study aimed to explore the efficacy and safety of remimazolam versus propofol for the induction and maintenance of general anesthesia in preschool-age children undergoing elective surgery.

**Methods and analysis:** In this multicenter, randomized, single-blind, positive-controlled non-inferior clinical trial, one hundred ninety-two children aged 3–6 years will be randomly allocated as a 3:1 ratio into two groups: Group R with an intravenous dose of remimazolam 0.3 mg/kg for the induction of anesthesia followed by a constant infusion rate of remimazolam 1–3 mg/kg/h to maintain anesthesia, and Group P with an intravenous dose of propofol 2.5 mg/kg for the induction of anesthesia followed by a constant infusion rate of propofol 4–12 mg/kg/h to maintain anesthesia. The primary outcome will be the rate of the successful induction and maintenance of anesthesia. The secondary outcomes will include the time to LoC, the Bispectral Index (BIS) value, awakening time, extubation time, post-anesthesia care unit (PACU) discharge time, usage of additional sedative drugs during the induction period, usage of remedial drugs in PACU, emergence delirium, pain in PACU, behavior scores at day 3 after surgery, parental and anesthesiologists’ satisfaction, and adverse events.

**Ethics and dissemination:** This study has been approved by the ethics review boards at all participating hospitals. The Ethics Committee of the Second Affiliated Hospital and Yuying Children’s Hospital of Wenzhou Medical University (Reference No. LCKY 2020-380, November 13, 2020) is the central ethics committee.

**Clinical Trial Registration:** Chinese clinical trial registry, identifier ChiCTR2100053468 (22 November 2021)

## 1 Introduction

As undergoing invasive operation and surgery is a stressful and traumatic experience for children, general anesthesia has been the anesthesia regimen of choice in pediatric patients (Cavuoto et al., 2014). The previous study has explored the tight relationship between inhalational anesthesia and postoperative behavioral disturbance incarnated as emergence agitation (EA) or emergence delirium (ED) in pediatric patients (Urits et al., 2020). The mask induction of volatile anesthetic could further aggravate anxiety, and even bring about long-term psychological trauma. Despite the fact that the establishment of peripheral venous access is an invasive procedure before anesthesia induction, fear and anxiety is significantly reduced when accompanied by the parents and some children can even be very cooperative. Therefore, the mainstream view is that total intravenous anesthesia (TIVA) was preferred over inhalational anesthesia (Lauder, 2015).

Among all intravenous anesthetic agents, propofol is the most popular sedative agent used for intravenous induction and maintaining the effects of general anesthesia in pediatric patients by virtue of its advantages in rapid onset, short duration of action, impressive speed and quality of recovery, minimization of ED, decrement of laryngospasm and bronchospasm, and curtailment of postoperative nausea and vomiting (PONV) (Chidambaran et al., 2015; Gentili, 2020). Despite these positive attributes, shortcomings comprising pain on intravenous injection, anaphylaxis, mitochondrial diseases, and propofol-related infusion syndrome (PRIS) have selectively restricted its further application in pediatric patients (Chidambaran et al., 2015). The important point to remember is that pain on intravenous injection of propofol has become the most common adverse effect. Although addition of the local anesthetic lignocaine to propofol in conjunction with use of a propofol emulsion containing medium and long chain triglycerides alleviates the pain, about 35% of pediatric patients still experience pain (Varghese et al., 2010; Jalota et al., 2011). Children who receive a single dose of propofol 1–3 mg/kg for induction usually experience brief apnea, which if not addressed promptly is most likely to develop severe hypoxemia. Moreover, propofol is mainly biotransformed by glucuronidation in the liver and excreted by the kidneys (Al-Jahdari et al., 2006), and the hepatic and renal functions in children are not yet mature enough to facilitate this process. In order to provide safer and smoother anesthesia for pediatric patients, much attention has been devoted to the search for more desirable sedatives.

Remimazolam, a novel ultra-short-acting benzodiazepine, is currently approved as an intravenous agent in multiple countries for use in general anesthesia, procedural sedation, and longer-term sedation in adults, including intensive care (Keam, 2020). It has a high specificity and affinity for the brain benzodiazepine site on the gamma-aminobutyric acid type A (GABAA) receptor to potentiate the action of the inhibitory neurotransmitter without appreciable affinity for any of the other receptor sites (Kilpatrick et al., 2007; Keam, 2020). In contrast to midazolam, remimazolam as a typical ‘soft drug’ (Buchwald, 2020) has the same ester linkage as remifentanil, which leads to rapid biotransformation in the body by widespread tissue esterases to a pharmacologically inactive carboxylic acid metabolite (CNS7054) (Kilpatrick et al., 2007). As a result of its organ-independent metabolism profile, the pharmacokinetics of remimazolam are not subject to any stage of renal impairment, and mild or moderate hepatic impairment (Stöhr et al., 2021). Remimazolam was clinically demonstrated to produce high clearance (CL), faster onset and offset rates than midazolam, and predictable sedation effects over a wide dose range without significant cardiovascular or respiratory depression in two phases I studies (Antonik et al., 2012; Schüttler et al., 2020), in which volunteers received remimazolam as intravenous single ascending dose administration or constant intravenous infusion, respectively. Pharmacokinetic/pharmacodynamic modeling supports the observation of time-dependent change in remimazolam CL that is not related to cumulative remimazolam dose within 24 h of continuous dosing (Zhou et al., 2020). The sedative effect of remimazolam is completely antagonized by flumazenil as a benzodiazepine receptor antagonist (Chen et al., 2020b). These characteristics may reduce the risk of oversedation and rebound sedation under these circumstances of chronic infusions or higher doses in pediatric patients.

Considering the properties of non-irritating, rapidly-acting, short half-life, acceptable and predictable level of sedation, fast emergence from anesthesia, non-dependence on liver and kidney metabolism, minimal residual sedation, inappreciable side effects associated especially with cardiovascular and respiratory depression at usual dosage, and available specific antagonist, remimazolam appears to be suitable for use in children. To date, a search of the literature revealed two case reports of successful remimazolam-based anesthesia for a pediatric patient—one with Duchenne muscular dystrophy (Horikoshi et al., 2021) and the other receiving general anesthesia under intraoperative direct cortical MEP monitoring due to supratentorial glioma (Kamata et al., 2022)— and a clinical trial of remimazolam for sedation in pediatric patients undergoing magnetic resonance imaging (Shioji et al., 2022). Nevertheless, there has been a notable deficiency of empirical randomized clinical trials focusing particularly on the use of remimazolam for general anesthesia in pediatric patients. The primary purpose of this study will be to obtain data that will help to explore the efficacy and safety of remimazolam versus propofol for induction and maintenance of general anesthesia in preschool-age children undergoing elective surgery.

## 2 Methods

### 2.1 Study design

This trial will be designed as a multicenter, single-blind, non-inferior randomized clinical trial to assess the efficacy and safety of remimazolam versus propofol for the induction and maintenance of general anesthesia in children undergoing elective surgery conducted at three Chinese medical centers (The Second Affiliated Hospital and Yuying Children’s Hospital of Wenzhou Medical University, the Children’s Hospital of Chongqing Medical University, Hunan Children’s Hospital). The protocol was approved by the ethics review boards at all participating hospitals and registered with the Chinese Clinical Trial Registry (ChiCTR2100053468, 22 November 2021). The trial is currently in the recruitment phase. Before inclusion, written informed consent will be obtained from their parents or legally authorized representatives of pediatric patients.

### 2.2 Participants

#### 2.2.1 Inclusion criteria

·Aged 3–6 years.
·American Society of Anesthesiologists (ASA) physical status I or II.
·A normal range of weight and body mass index (BMI) 14–25 kg/m^2^.
·Booked for elective surgery (except cardiothoracic, neurosurgical or hepatic surgery) under general anesthesia with endotracheal intubation.
·The informed consent signed by parents or legally authorized representatives.

#### 2.2.2 Exclusion criteria

·Requiring special care or supervision of court/social welfare institutions.
·Undergoing general anesthesia within 14 days before randomization.
·Participating in clinical trials of other drugs within 3 months prior to screening.
·In combination with other forms of anesthesia (such as inhalation anesthesia, epidural anesthesia, subarachnoid anesthesia, regional block, etc.) between entry into the operating room until the end of the operation.
·Existing other serious illnesses with severe cardiovascular diseases, critical anemia, liver dysfunction (aspartate aminotransferase (AST) and/or alanine aminotransferase (ALT) ≥1.5 upper limit of normal (ULN), total bilirubin (TBIL) ≥ULN), renal dysfunction (serum creatinine and/or urea nitrogen > ULN), neurological disorders including cognitive dysfunction or epilepsy or acute angle-closure glaucoma (AAGG).
·Complicated with the difficulty of respiratory management (grade III or IV Modified Mallampati Score) or preexisting abnormal medication behavior.
·Suspected to be allergic or contraindicated to benzodiazepines, propofol or drugs specified in this trial (sufentanil citrate, cis-atracurium, remifentanil hydrochloride, ketorolac tromethamine, neostigmine mesylate or atropine sulfate).
·Not suitable for participating in this clinical trial judged by the investigator.

### 2.3 Randomization and masking

After giving consent, eligible children will be randomized by an interactive web response system (IWRS) at a 3:1 ratio into the remimazolam group (Group R) or propofol group (Group P). Randomization will be stratified by centers to limit baseline imbalance between treatment groups. All study drugs will be prepared by a designated researcher with a stock solution of propofol (10 mg/mL, Fresenius Kabi Austria GmbH, Graz, Austria) and remimazolam tosylate (36mg, Jiangsu Hengrui Pharma Corporation, China) dissolved in sterile saline solution 0.9% to a concentration of 1 mg/mL. In consideration of the obvious difference in drug color and dose adjustments, only the participants will be blinded to the treatments throughout the study. Trained outcome evaluators blinded to group allocation will be assigned to assess the prescribed endpoints after the operation.

### 2.4 Study procedures

#### 2.4.1 Pre-anesthesia visit

During the routine preoperative anesthesia visit, an anesthetist will collect demographic data, vital signs, medical history, concomitant medication and preoperative laboratory tests (complete blood count, coagulation and serum biochemical), and carry out a physical examination for each child. Their parents or legally authorized representatives will give their informed consent after being advised on the purpose of the study, associated risks and benefits.

#### 2.4.2 Induction of general anesthesia

Children’s preoperative fasting status will be confirmed to comply with an international multidisciplinary consensus statement on fasting before procedural (Green et al., 2020). No children will receive any preoperative medication. The baseline vital signs and the level of alertness will be recorded in a quiet area accompanied by their parents or legally authorized representatives before arrival at the operating room; the vital signs at the pre-anesthesia visit will be considered as baseline for children who could not cooperate. We will use the Modified Observer’s Assessment of Alertness/Sedation scale (MOAA/S) (Klockars et al., 2006; Jung et al., 2019) (Supplementary Table S1)—a valid tool in the measurement of sedation levels for children by observing their response to various stimuli to evaluate the subject’s level of sedation. We will consider a MOAAS score ≤1 as the sign of loss of consciousness (LoC). In the specific assessment procedure, we will start intermittent gentle shoulder shaking immediately after the loss of the eyelash reflex, and no response will indicate LoC.

Before anesthesia induction, peripheral venous access will be established and the following vital signs will be monitored throughout the study: continuous electrocardiogram (ECG), heart rate (HR), respiratory rate (RR), pulse oxygen saturation (SpO_2_), and non-invasive blood pressure (NIBP) which will be adjusted by measuring at 1-min intervals until achieving endotracheal intubation and every 5min thereafter. The Bispectral Index (BIS, Aspect Medical Systems-Covidien) pediatric sensor strip will be applied on the forehead of every child, and the BIS value will be recorded before the dose, at LoC, after tracheal intubation and thereafter at 5-min intervals. Subsequently, all pediatric patients will be pre-oxygenated for several minutes in 100% oxygen at a flow rate of 3 L/min followed by a single dose of fentanyl 3 μg/kg (Yichang Humanwell Pharmaceutical, China) intravenously. About 3 min later, all pediatric patients will randomly receive an initial intravenous dose of remimazolam 0.3 mg/kg in Group R or propofol 2.5 mg/kg in Group P for the induction of anesthesia, the injection time ≤60s. The investigator will administer an additional intravenous dose of remimazolam 0.1 mg/kg in Group R or propofol 1.0 mg/kg in Group P under such condition that the child will not achieve the loss of consciousness (LoC: MOAA/S score ≤1) within 60s, the injection time ≤30s. In both groups, the additional dose will be limited to single-use only. If LoC still does not occur within 1 min after the additional dose, propofol 1.0 mg/kg as the remedial sedative drug will be administered repeatedly by intravenous injection until MOAA/S score ≤1, in which general anesthesia will be maintained using isoflurane inhalation instead of propofol or remimazolam.

After reaching LoC without the use of the remedial sedative drug, the researcher will immediately begin taking an intravenous infusion of remimazolam at an initial rate of 2 mg/kg/h in Group R or propofol at an initial rate of 8 mg/kg/h in Group P and remifentanil (Yichang Humanwell Pharmaceutical, China) at an initiation rate of 0.25 μg/kg/min in both groups, while administering cis-atracurium 0.2 mg/kg intravenously. Tracheal intubation will be facilitated to provide assisted ventilation after approximately 3 min.

#### 2.4.3 Maintenance of general anesthesia

After tracheal intubation is completed, parameters of mechanical ventilation using volume-controlled or pressure-controlled ventilation mode with 50% oxygen/air mixture will be set according to age and ideal body weight to maintain PetCO2 between 35 and 45 mmHg. Anesthesia will be maintained with a constant infusion rate of remimazolam 1–3 mg/kg/h in Group R or propofol 4–12 mg/kg/h in Group P, and remifentanil 0.1–0.5 μg/kg/min in both groups.

The infusion rate of drugs will be adjusted within the prescribed maintenance dose range in the context of the changing intensity of surgical stimuli as primary guidance and the BIS value as secondary guidance to maintain an appropriate depth of anesthesia. Remedial sedative and/or analgesic drugs will be given to children suffering from an inadequate depth of anesthesia defined as the appearance of at least one of the following signs: patient movement, sweating, tears, hypertension and tachycardia (systolic blood pressure (SBP) and HR exceeding 25% of the baseline value for more than 5min), or clear and substantial elevations in SBP or HR, followed by discontinuing the investigational drugs and switching to continuous inhalation of isoflurane. The choice of remedial sedative and/or analgesic medications and dosage will be determined by the investigator. If hemodynamic instability persists in either group, additional vasoactive agents (e.g., esmolol or urapidil) will be administered at the investigator’s discretion. In both groups hypotension (SBP <70 mmHg + (2 x age in years) for a further 5 min) and bradycardia (HR < 70 bpm for a further 5 min) will be treated with ephedrine/phenylephrine and atropine, respectively, and the investigator will decide whether to discontinue the study drugs If necessary.

Fentanyl 1 μg/kg as an additional dose will be given as needed each time intravenously prior to the anticipated appearance of the significantly larger intensity of surgical stimuli during surgery, and the total intraoperative additional fentanyl dose will be limited to a maximum of 5 ug/kg. Intraoperative muscle relaxation will be given as needed by administration of additional doses of cis-atracurium 0.05 mg/kg each time, aiming to serve surgical needs or maintain effective mechanical ventilation, and no additional muscle relaxants will be administered within approximately 30 min prior to termination of surgery. Ketorolac tromethamine 0.5 mg/kg will be administered intravenously in a usual manner approximately 30 min before termination of surgery, and local infiltration anesthesia, nerve block or sacral anesthesia will be implemented if clinically indicated after surgery for postoperative analgesia. The type, concentration, and dose of local anesthetic will be at the discretion of the investigator. Residual muscle relaxation will be antagonized as required with neostigmine 0.03 mg/kg and atropine 0.015 mg/kg at the end of surgery.

#### 2.4.4 Recovery from general anesthesia

All the drugs will be discontinued at the end of the surgery. A child who has regular spontaneous breathing and recovery of airway reflexes will be extubated and transferred to the post-anesthesia care unit (PACU) for continuing regular anesthesia monitoring, where an attending anesthesiologist who will be unaware of trial grouping will assess the level of sedation, ED, and pain.

After removal of the investigational drugs, MOAA/S scores will be assessed every 5 min for at least 40 min until 3 consecutive MOAA/S scores of 5. In group R, as long as no arousal occurs after 1 h from the end of remimazolam administration, 0.2 mg flumazenil will be given and a dose of 0.1 mg will be repeated as needed. ED and pain in PACU individually will be evaluated using the Pediatric Anesthesia Emergence Delirium (PAED) scale (Sikich and Lerman, 2004) (Supplementary Table S2)—a reliable tool in the diagnosis of ED and the face, legs, activity, cry and consolability (FLACC) scale (Crellin et al., 2018) (Supplementary Table S3)—a validated pain measurement tool for children aged 2 months to 7 years. Both scales will be recorded every 5 min for at least 30 min after the arrival of PACU. PAED scores ≥10 will be considered a diagnosis of ED and PAED scores ≥15 will be defined as a severe ED. A child considered to have developed delirium or moderate and severe pain (FLACC scale >3) will be treated with remedial sedatives and/or analgesic drugs (e.g., propofol or fentanyl) at the attending anesthesiologist’s discretion. The modified Aldrete score will be recorded every 3 min after 3 consecutive MOAA/S scores of 5, and the child will be admitted to discharge from the PACU until 2 consecutive modified Aldrete scores ≥9.

#### 2.4.5 Postoperative follow-up

Regular postoperative follow-up will be carried out for the included pediatric patients *via* telephone or subsequent visit at least 24 h after the end of the surgery. Collected data will include vital signs, concomitant medication, and postoperative laboratory tests (Complete blood count, coagulation and serum biochemical) compared with preoperative data to assess the safety of the investigational drugs and to document adverse events. Furthermore, participants will be followed up with again on day 3 postoperative with parents or legally authorized representatives asked by telephone to complete the evaluation of any negative behavior changes using the Post-Hospitalization Behavior Questionnaire for Ambulatory Surgery (PHBQ-AS) (Jenkins et al., 2015) (Supplementary Table S4)—a parental report measure used to assess negative behavior change after hospitalization, consisting of 11 items on 1-5 score. Each item will have a ‘Not Applicable’ option scoring 3 to avoid problems with missing data. The PHBQ-AS score will be calculated for each respondent as the mean score of all individual items answered on the questionnaire. A score above 3 will indicate the presence of negative behavioral change, a score equal to 3 will indicate no behavioral change, and a score below 3 will indicate an improvement in behavior.

### 2.5 Outcomes

The primary endpoint will be the incidence of the successful induction and maintenance of anesthesia. Successful anesthesia induction will be defined as LoC being reached without the use of remedial sedative drugs during the induction period. Successful anesthesia maintenance will be defined as neither need for rescue sedative medication nor discontinuity of investigational drugs during the maintenance period.

The secondary endpoints will include 1) time to LoC: the time interval from an initial dose of investigational drugs to the first MOAA/S score ≤1; 2) usage of additional sedative drugs during the induction period; 3) the BIS value before dose, at LoC, after tracheal intubation and during the maintenance of anesthesia (LoC to end of the investigational drugs); 4) awakening time: the time interval from the end of investigational drugs to the first of 3 consecutive MOAA/S scores of 5; 5) extubation time: the time interval from discontinuation of investigational drugs to extubation; 6) the incidence of ED, moderate and severe pain (FLACC scale >3) in PACU; 7) usage of remedial drugs in PACU; 8) PACU discharge time: the time interval from the end of investigational drugs to the first of 2 consecutive modified Aldrete score ≥9; 9) parental and anesthesiologists satisfaction; and 10) the incidence of negative behavior change at day 3 after surgery.

Safety endpoints will include frequency, causality, and severity of adverse events (AEs) in each treatment arm. Grading of AEs will be according to Common Terminology for Adverse Event (CTCAE), Version 5.0 criteria. Injection site pain, fluctuations in blood pressure or heart rate, and the use of vasoactive agents will be recorded. Postoperative nausea and vomiting (PONV), delayed awakening from anesthesia, and respiratory depression will also be collected.

Detailed information about surgery will be collected: name of surgery, length of surgery, intraoperative bleed loss, the total maintenance dose of study drugs (remimazolam or propofol) and remifentanil, total cumulative additional consumption, frequency of fentanyl and cis-atracurium, and usage of perioperative concomitant medication.

### 2.6 Sample size and statistical power

Sample size estimates were based on a non-inferiority design. Considering a one-sided type I error α = 0.025, a type II error *ß* = 0.10, a non-inferiority margin δ = − 8%, a proportion of the successful anesthesia induction and maintenance of 98% in both treatment groups, and an intervention: control ratio of 3: 1. One hundred seventy-one pediatric patients were calculated using PASS Version 15.0 (NCSS, United States). Together with an expected loss to follow-up of 10%, we required a total of 192 pediatric patients (144 in Group R, 48 in Group P). The proportion of the successful anesthesia induction and maintenance of 98% in both treatment groups was set under pre-test in pediatric patients and previous studies in adults (Doi et al., 2020; Sheng et al., 2020; Dai et al., 2021).

### 2.7 Statistical methods

All randomized patients who will receive at least one treatment specified in the trial will be included in the full analysis set (FAS). Continuous variables will be tested for normality using a Shapiro-Wilk normality test. Demographics and all endpoints will be presented as numbers and percentages (dichotomous variables), means and standard deviations (normally distributed continuous variables), and medians and interquartile ranges (non-normally distributed continuous variables). Comparison between groups of all dichotomous endpoints will be analyzed with the χ2 test, continuity correction χ2 test or Fisher’s exact test whenever it is appropriate. We will compare the normally distributed continuous endpoints using independent samples *t*-test and calculate the difference (and 95% CI of the difference) between means. Non-normally distributed continuous endpoints will be compared using Mann–Whitney *U* test, and the difference (and 95% CI of the difference) between medians will be estimated using Hodges–Lehmann estimators. Non-inferiority will be assumed if the lower limit of a two-sided 95% CI of the difference between Group R and Group P in the percentage of participants meeting the primary endpoints exceeds the non-inferiority margin. Missing data will be considered to be missing at random without data imputation. Available case analysis will be implemented to handle missing data such as PHBQ-AS score measured during follow-up. *p*-value < 0.05 will be considered statistically significant. Data analysis will be performed using SPSS version 26.0 for Windows (SPSS Inc., Chicago, IL, United States).

## 3 Discussion

Pediatric anesthesia has consistently been seen as a conundrum for anesthesiologists in various fields of surgery and diagnostic procedures due to a far more stressful response to surgery or invasive operation in the pediatric population than in adults. As a result, it has been well acknowledged that general anesthesia is the preferred anesthetic management option for the pediatric population, with increasing utilization of agents such as propofol, midazolam and dexmedetomidine (Lauder, 2015), and TIVA seems to be more applicable than inhalational anesthesia. Remimazolam, a member of benzodiazepines, exerts its clinical effect by means of highly selective allosteric modulation of the GABAA receptor and shares some properties with both its parent compounds, midazolam and remifentanil. During this past decade, it was widely used as an intravenous agent for general anesthesia, procedural sedation and intensive care unit (ICU) sedation in adults. It exhibited distinctive and amazing advantages concerning pharmacological and pharmacokinetic properties over propofol, midazolam and dexmedetomidine. The findings of several phase III studies (Rex et al., 2018; Pastis et al., 2019; Rex et al., 2021) in sedation for endoscopy have documented a higher procedural success rate, more rapid onset of action, faster time to return to wakefulness from sedation in remimazolam group than in midazolam group, without significant differences regarding safety or adverse effects. A phase III study (Chen et al., 2022) comparing a slow initial dose of remimazolam versus dexmedetomidine for 10 min followed by appropriate maintenance dose in conjunction with continuous infusion of remifentanil to maintain MOAA/S scale <3 for 146 outpatients undergoing FB simultaneously reported that remimazolam-remifentanil had a non-inferior efficacy, better time metrics and hemodynamic stability compared to dexmedetomidine-remifentanil. Moreover, several phase II or III studies with propofol-control have been conducted on adult patients receiving endoscopic procedures (Chen et al., 2020; Chen et al., 2021; Zhang et al., 2021), in which remimazolam showed non-inferior procedural success rate, better respiratory management and hemodynamic stability, and lower incidence of injection site pain than propofol. A Japanese phase IIb/III trial (Doi et al., 2020) evaluating the non-inferior efficacy and safety of remimazolam versus propofol for the induction and maintenance of general anesthesia in adult patients undergoing elective surgery reported a similar pharmacodynamic profile to the above endoscopic therapy studies.

Hence, the striking features of remimazolam include faster onset and offset rates, a shorter half-life than midazolam and dexmedetomidine, lower accumulation and risk of cardiovascular and respiratory depression than propofol, and availability of a reversal agent, which should be sufficient to increase the accuracy of the hypothesis—non-inferior efficacy and more limited AEs of this drug in induction and maintenance of general anesthesia in preschool-age children than propofol. Particularly, it should be suitable for short-term operations and day-surgery where the child needs to be discharged from the hospital as early as possible.

In our research, we will include 192 children aged 3–6 years to explore the efficacy and safety of remimazolam in preschool-age children in consideration of the large variation in physiological changes in children which leads to large differences in pharmacokinetic processes, and the difficulty in establishing peripheral venous access preoperatively in children under 3 years of age. We choose propofol as the control drug instead of midazolam or dexmedetomidine, mainly because propofol is the most commonly used intravenous agent for the induction and maintenance of anesthesia, procedural, and critical care sedation in pediatric patients (Chidambaran et al., 2015). Furthermore, midazolam and dexmedetomidine have always been used as premedications for pediatric patients to relieve anxiety and distress, enhance compliance with anesthesia induction, and reduce the incidence of ED (Lang et al., 2020).

We will make continual adjustments to the infusion rate of investigational drugs in the context of the changing intensity of surgical stimuli as primary guidance and the BIS value as secondary guidance to maintain an optimum depth of anesthesia. Drawing on the “pressure, rate, sweating and tears” (PRST) scoring system (Russell, 1993), light anesthesia is defined as the occurrence of at least one of the following signs: patient movement, sweating, tears, hypertension and tachycardia (SBP and HR exceeding 25% (Lei et al., 2017) of the baseline value for more than 5min), or clear and substantial elevations in SBP or HR. Hypotension is SBP <70 mmHg + (2 x age in years) for a further 5 min. This is in accordance with previously described guidelines for hypotension by the Pediatric Advanced Life Support Provider Manual (Haque and Zaritsky, 2007). Bradycardia is usually defined as a heart rate below the lowest normal value for age. Thus, we define bradycardia as HR < 70 bpm for a further 5 min in children aged 3–6 years with reference to normal values for age (Baruteau et al., 2016). As everyone knows, the vagal excitatory reflex and harm may be magnified at the transient and extreme surgical stimuli. Consequently, it is the few minutes of SBP and HR fluctuations that matter. We then define hypertension, tachycardia, hypotension and bradycardia sustained for at least 5 cumulative minutes. It is worth noting that the appearance of clear and substantial elevations in SBP or HR must be immediately adjusted and processed by using remedial drugs.

The electroencephalogram-based (EEG-based) systems have been used for assessing the appropriate depth of general anesthesia. However, the EEG in younger children is increasingly different from that of adults, and the currently available processed EEG devices (BIS Monitor, the Patient State Index (PSI) of the SEDLine brain function monitor or the Narcotrend index) are based on adult EEG data and may not directly apply to the pediatric population accompanying age-related differences (Grasso et al., 2021). Some studies have shown that the BIS value during anesthesia with remimazolam was reported to be higher than with propofol (Doi et al., 2020), even in some cases mean intraoperative BIS values were reported to be over 60 in five out of thirty patients who underwent breast surgery under general anesthesia with remimazolam, and mean intraoperative PSI values were >50 in eight patients out of the thirty patients (Shirozu et al., 2022). Likewise, it was reported that the single doses of 0.075–0.3 mg/kg in healthy adults resulted in deep sedation (MOAA/S score ≤1) and mean BIS values of 60–70 after dosing (Antonik et al., 2012). Our pre-test data in pediatric patients under general anesthesia with remimazolam showed a similar experimental result—satisfactory hypnotic efficacy but mean BIS values of over 60 throughout the period of anesthesia induction and maintenance. Although PSI showed a definite dose-response relationship under remimazolam bolus injection, PSI does not appear to be a reliable surrogate of LoC (Chae et al., 2022). The correlation between the Narcotrend Index and MOAA/S score was relatively weak and inconsistent, suggesting that the Narcotrend Index might be less suitable for monitoring the sedative effect if remimazolam is administered alone (Eisenried et al., 2020). Accordingly, the BIS will be monitored continuously during surgery as secondary guidance instead of primary guidance to maintain an optimum depth of anesthesia in the protocol. It can indeed be seen that lack of reliable depth of anesthesia monitoring might keep pediatric patients under the potential risk of deep anesthesia or intraoperative awareness during the procedure. This may increase the incidence of a negative impact on short and long-term outcomes. A semistructured Brice questionnaire could be used in children aged older than 5 years to evaluate the incidence of suspected and true awareness, but regrettably, does not apply to the children within this protocol aged less than 5 years because of their inability to accurately answer the questions asked in the questionnaire (Lee et al., 2021). Therefore, the main purpose of PAED scale and PHBQ-AS on day 3 postoperatively is to remedy this imperfection to some extent. Pain is a key confounding factor in the diagnosis of ED and the evaluation of negative behavior change postoperatively, and so regional anesthetic techniques (local infiltration anesthesia, nerve block or sacral anesthesia) could be admitted to offer control of postoperative pain after the end of surgery in this protocol. Both FLACC scale and PAED scale will be assessed at the same time to avoid conflating symptoms and actions secondary to postoperative pain with signs and symptoms of ED mainly owing to the remarkable similarities between the two clinical symptoms. We will perform the PHBQ-AS on day 3 postoperatively to quantify the apparent alleviation in pain and anxiety for most children discharged from a hospital.

There are several similarities in terms of pharmacological and pharmacokinetic characterization between remimazolam and midazolam commonly given as intraoperative or preoperative agents for anxiety control as well as preventive therapy for ED in children (Urits et al., 2020). Other interesting findings have shown remimazolam besylate, compared with dexmedetomidine, was equally effective in relieving agitation in older patients aged ≥70 years following orthopedic surgery (Deng et al., 2022) and remimazolam provided a non-inferiority quality of recovery (QoR) on postoperative day 1 to propofol for general anesthesia in female patients aged 20–65 years undergoing thyroid surgery (Choi et al., 2022). The assessment of QoR on day 1 postoperatively depends on the QoR-15 questionnaire, which is a global measure of recovery after surgery. These outcomes should contribute to the holding of another hypothesis—remimazolam applied on induction and maintenance of general anesthesia could be identically effective in decreasing the incidence of ED and negative behaviors after hospitalization in preschool-age children to propofol.
