# Supplementary material for: The safety and efficacy of remimazolam tosylate for induction and maintenance of general anesthesia in pediatric patients undergoing elective surgery: Study protocol for a multicenter, randomized, single-blind, positive-controlled clinical trial
Source: Front Pharmacol. 2023 Feb 10;14:1090608. doi: 10.3389/fphar.2023.1090608 (PMC9950936; doi:10.3389/fphar.2023.1090608)
Supplement: Supplementary file 1 [file Table1.docx]

**Table S1** Modified Observer's Assessment of Alertness/Sedation (MOAA/S) scale

| Score | Responsiveness |
| --- | --- |
| 5 | Responds readily to name spoken in normal tone |
| 4 | Lethargic response to name spoken in normal tone |
| 3 | Responds only after name is called loudly and/ or repeatedly |
| 2 | Responds only after mild prodding or shaking |
| 1 | Responds only after painful trapezius squeeze |
| 0 | Does not respond to painful trapezius squeeze |
