# Supplementary material for: The safety and efficacy of remimazolam tosylate for induction and maintenance of general anesthesia in pediatric patients undergoing elective surgery: Study protocol for a multicenter, randomized, single-blind, positive-controlled clinical trial
Source: Front Pharmacol. 2023 Feb 10;14:1090608. doi: 10.3389/fphar.2023.1090608 (PMC9950936; doi:10.3389/fphar.2023.1090608)
Supplement: Supplementary file 2 [file Table2.docx]

**Table S2** Pediatric anesthesia emergence delirium (PAED) scale

| Behavior | | Not at all | Just a little | Quite a bit | Very much | Extremely |
| --- | --- | --- | --- | --- | --- | --- |
| Makes eye contact | | 4 | 3 | 2 | 1 | 0 |
| Actions are purposeful | 4 | 3 | 2 | 1 | 0 |  |
| Aware of surroundings | 4 | 3 | 2 | 1 | 0 |  |
| Restless | 0 | 1 | 2 | 3 | 4 |  |
| Inconsolable | 0 | 1 | 2 | 3 | 4 |  |

1—Calm; 2—not calm but could be easily consoled; 3—moderately agitated or restless and not easily calmed; 4—combative, excited, thrashing around.
