# Supplementary material for: The safety and efficacy of remimazolam tosylate for induction and maintenance of general anesthesia in pediatric patients undergoing elective surgery: Study protocol for a multicenter, randomized, single-blind, positive-controlled clinical trial
Source: Front Pharmacol. 2023 Feb 10;14:1090608. doi: 10.3389/fphar.2023.1090608 (PMC9950936; doi:10.3389/fphar.2023.1090608)
Supplement: Supplementary file 3 [file Table3.docx]

**Table S3** Face, Legs, Activity, Cry and Consolability (FLACC) scale

| categories | items | Score |
| --- | --- | --- |
| Face | No particular expression or smile | 0 |
|  | Occasional grimace or frown; withdrawn, disinterested | 1 |
|  | Frequent to constant frown, clenched jaw, quivering chin | 2 |
| Legs | Normal position or relaxed | 0 |
|  | Uneasy, restless, tense | 1 |
|  | Kicking or legs drawn up | 2 |
| Activity | Lying quietly, normal position, moves easily | 0 |
|  | Squirming, shifting back and forth, tense | 1 |
|  | Arched, rigid, or jerking | 2 |
| Cry | No cry (awake or asleep) | 0 |
|  | Moans or whimpers, occasional complaint | 1 |
|  | Crying steadily, screams or sobs; frequent complaints | 2 |
| Consolability | Content, relaxed | 0 |
|  | Reassured by occasional touching, hugging, or being talked to; distractable | 1 |
|  | Difficult to console or comfort | 2 |

Interpreting the total Behavioral Score: 0 relaxed and comfortable, 1–3 mild discomfort, 4–6 moderate pain, 7–10 severe discomfort or pain or both.
