# Supplementary material for: The safety and efficacy of remimazolam tosylate for induction and maintenance of general anesthesia in pediatric patients undergoing elective surgery: Study protocol for a multicenter, randomized, single-blind, positive-controlled clinical trial
Source: Front Pharmacol. 2023 Feb 10;14:1090608. doi: 10.3389/fphar.2023.1090608 (PMC9950936; doi:10.3389/fphar.2023.1090608)
Supplement: Supplementary file 4 [file Table4.docx]

**Table S4** Post Hospitalization Behavior Questionnaire for Ambulatory Surgery（PHBQ-AS）

| 1. Does your child make a fuss about eating?  2. Does your child spend time just sitting or lying and doing nothing?  3. Is your child uninterested in what goes on around him (or her)?  4. Does your child get upset when you leave him (or her) alone for a few minutes?  5. Does your child need a lot of help doing things?  6. Is it difficult to get your child interested in doing things (like playing games with toys)?  7. Does your child have temper tantrums?  8. Is it difficult to get your child to talk to you?  9. Does your child have bad dreams at night or wake up and cry?  10. Does your child have trouble getting to sleep at night?  11. Does your child have a poor appetite? |
| --- |

Each item is scored as follows: 1 much less than before, 2 less than before, 3 same as before / not applicable, 4 more than before, 5 much more than before.
